# Verschlechterung der Lebensqualität von Patienten mit Morbus Hodgkin in den folgenden 5 Jahren nach abgeschlossener Therapie: Langzeitdaten aus den Studien HD13, HD14 und HD15

**DOI:** 10.1007/s00066-021-01755-8

**Published:** 2021-03-22

**Authors:** Dirk Vordermark

**Affiliations:** Universitätsklinik für Strahlentherapie, Universitätsklinikum Halle/Saale, Ernst-Grube-Str. 40, 06120 Halle/Saale, Deutschland

## Hintergrund

Der stadien- und risikoadaptierte Einsatz multimodaler Therapiekonzepte erzielt beim Morbus Hodgkin bekanntermaßen hohe Heilungsraten. Die Langzeitüberlebenden sind jedoch in vielen Bereichen ihrer Lebensqualität beeinträchtigt. Dies beinhaltet spezifische Symptomkomplexe wie Fatigue, aber auch Probleme bei der sozialen und beruflichen Integration der häufig bei Therapie noch sehr jungen und somit im Langzeitverlauf immer noch jungen Patienten. Die Therapieoptimierungsstudien der Deutschen Hodgkin Studiengruppe (DHSG) zielten auf eine Verbesserung des Profils zwischen onkologischer Tumorkontrolle (z. B. gemessen als progressionsfreies Überleben) und Spätfolgen der Therapie ab, z. B. durch Weiterentwicklung der Chemotherapiekonzepte und Modifikation der Strahlentherapie bezüglich Zielvolumen und Dosierung, teilweise auch durch einen Verzicht auf die Radiotherapie. Aktuell wurde nun ein sehr umfangreicher Datensatz zur Lebensqualität im Langzeitverlauf bis fünf Jahre nach Therapieende aus den Studien HD13, HD14 und HD15 publiziert, der eine differenzierte Betrachtung der Entwicklung definierter Bereiche der Lebensqualität wie auch die Identifikation von Risikofaktoren einschließlich des Zusammenhangs mit einzelnen Therapiekonzepten erlaubt.

## Patienten und Methoden

Berichtet werden die Daten von 4215 Patienten aus der Studiengeneration HD13 bis HD15 der DHSG mit Beantwortung des Lebensqualitätsfragebogens zu mindestens einem Zeitpunkt. Eingeschlossen wurden Patienten der Altersgruppe von 18 bis 60 Jahren (bei Therapie), unabhängig vom Remissionsstatus. Die Studie HD13 verglich in der Gruppe der günstigen Frühstadien (Ann Arbor I oder II, ohne Risikofaktoren) vier Arme der Chemotherapie (ABVD, AVD, ABV, AV; die zwei letztgenannten wurden frühzeitig geschlossen). Alle Patienten erhielten eine Involved-field-Radiotherapie mit 30 Gy [1]. Für den Arm ABVD wurde ein progressionsfreies 5‑Jahres-Überleben von 93 % erreicht, die anderen Arme waren schlechter. Die Studie HD14 untersuchte in der Gruppe der ungünstigen Frühstadien (I bis II mit definierten Risikofaktoren) die Chemotherapie mit 4 Zyklen ABVD im Vergleich zu 2 Zyklen ABVD + 2 Zyklen eskaliertem BEACOPP. Auch hier erhielten alle Patienten eine Involved-field-Radiotherapie mit 30 Gy [2]. Das progressionsfreie Überleben war mit 95 % signifikant besser nach 2 × ABVD + 2 × BEACOPP. In HD15 wurden drei Varianten von BEACOPP bei Patienten mit Stadien IIB (mit mediastinalem Bulk) bis IV verglichen. Die Strahlentherapie erfolgte in allen Armen nur auf PET-positive Reste > 2,5 cm nach Chemotherapie mit 30 Gy, dies betraf lediglich11 % der Patienten [3]. Der Arm mit 6 Zyklen BEACOPP-eskaliert erreichte das beste progressionsfreie Überleben nach 5 Jahren mit 90 %.

Für die aktuell präsentierte Analyse der Lebensqualität wurde der etablierte und validierte Fragebogen European Organization for Research and Treatment of Cancer (EORTC) Quality of Life Questionnaire (QLQ C30) verwendet. Die Befragung erfolgte zu Therapiebeginn, nach zwei bis vier Zyklen Chemotherapie, am Ende der Erstlinienbehandlung (Chemotherapie ± Radiotherapie) und dann zu den Nachsorgezeitpunkten bis fünf Jahre nach Therapie. Fünf Funktionsbereiche und neun Symptombereiche wurden ausgewertet und mit den alters- und geschlechtsadjustierten Werten der deutschen Normalbevölkerung verglichen. Unterschiede von mindestens 10 Punkten (auf der Skala von 0 bis 100) wurden gemäß der üblichen Kriterien als klinisch relevant bewertet.

## Ergebnisse

Die Rücklaufraten lagen zu Therapiebeginn bei etwa 80 %, im zweiten Jahr nach Therapie bei etwa 65 % und im fünften Jahr bei knapp 40 %. Starke Selektionseffekte (z. B. Überrepräsentation der Patienten mit weniger Belastung) konnten ausgeschlossen werden. Bei Diagnosestellung wurden in allen Stadiengruppen und Studien (am ausgeprägtesten in höheren Stadien/HD15) starke Belastungen in den Bereichen der emotionalen und sozialen Funktion und bei den Symptomen Fatigue, Schlafstörung und finanzielle Problemen gesehen. Symptome wie Dyspnoe und Appetitverlust waren nur in der mittleren und höheren Stadiengruppe (HD14, HD15) auffällig. Unter der Chemotherapie trat in den meisten Bereichen eine weitere Verschlechterung auf, am dramatischsten in der von den Autoren als „Triade“ bezeichneten Gruppe Fatigue, Rollenfunktion und soziale Funktion.

Im ersten Jahr nach Therapieende und teilweise auch noch bei längerer Nachbeobachtung traten signifikante Erholungen in allen Bereichen auf bis auf die körperliche Funktion. Im betrachteten 5‑Jahres-Zeitraum verblieb hier eine deutlich schlechtere Lebensqualität (mit einem klinisch relevanten Delta von 14 bis 22) in allen Funktionsskalen (kognitiv, emotional, sozial), ebenso bei den Symptomen Fatigue, Dyspnoe, Schlafstörung und finanzielle Probleme. Innerhalb der einzelnen Studien konnten keine Unterschiede in der Lebensqualität zwischen den einzelnen Therapiearmen mit unterschiedlichen Chemotherapiekonzepten detektiert werden.

Schlussfolgerungen der Autoren: Bei Langzeitüberlebenden nach Therapie eines Hodgkin-Lymphoms fällt eine umfangreiche und persistierende Verschlechterung der Lebensqualität auf, die weitgehend unabhängig vom gewählten Chemotherapiekonzept ist.

## Kommentar

Der Begriff der Lebensqualität ist in der Onkologie zunehmend aus der Mode gekommen und wird in den letzten Jahren vor allem durch das „patient-reported outcome“ (PRO) und dessen elektronische Messung ersetzt. Deren Berücksichtigung im Behandlungsablauf kann die Überlebensprognose der Patienten verbessern [4]. Beiden Konzepten ist gemein, dass die von den Patienten gemachten Angaben (in Papierform oder elektronisch auf einem Smartphone oder Tablet-PC) bei Verwendung validierter Fragebögen ohne eine weitere Zwischenbewertung durch medizinisches Personal in aussagekräftige Scores übersetzt werden.

Die Betrachtung der gesundheitsbezogenen Lebensqualität („health-related quality of life“ [HRQOL]) wird dadurch erschwert, dass sie multidimensional ist, also nicht durch einen, sondern durch eine Gruppe von Scores beschrieben wird. Das führt dazu, dass in der (radio-)onkologischen Literatur Lebensqualitätsstudien oft schwer lesbar sind, da unübersichtliche Tabellen generiert werden und durch multiples Testen „immer irgendetwas signifikant“ ist.

Die Situation der Langzeitüberlebenden nach Therapie des Hodgkin-Lymphoms steht schon lange im Fokus. Denn die Patienten sind überwiegend jung, die Therapien intensiv und die Heilungsraten hoch. Relevante Spätfolgen der Chemotherapie oder der Strahlentherapie wie Zweittumoren oder Kardiotoxizität können nach Jahren bis Jahrzehnten auftreten [5]. Eine Deeskalation der Therapie mit Absenkung der Spätfolgenrisiken bei Erhalt der onkologischen Therapieeffektivität steht deshalb im Fokus der Studienaktivitäten in den jetzt betrachteten erwachsenen Kollektiven. Im pädiatrischen Bereich wird noch stärker sogar die Inkaufnahme einer erhöhten frühen Rezidivrate (bei bestehender Salvage-Option) als akzeptabler Preis für vermiedene Spätfolgen diskutiert [6].

Die vorliegende Arbeit ist zunächst von der Methodik der Auswertung und Ergebnisdarstellung ein Meilenstein. Im Mittelpunkt steht der klinisch relevante Effekt, also das Ausmaß der Abweichung vom Mittelwert der Normalbevölkerung. Dieser wird – trotz der großen Datenmenge – stratifiziert nach Studie, Symptomatik und Zeitpunkt (sowie im Appendix nach Altersgruppe) anschaulich in farbcodierten Heatmaps dargestellt, die für den Strahlentherapeuten intuitiv sind und die „Hot Spots“ der Probleme schnell erkennbar machen.

Die Erkenntnis, dass die Patienten sich von den extremen Belastungen der Therapie zwar partiell erholen, aber in fast keinem Bereich in den ersten fünf Jahren wieder in die Nähe der Normalwerte kommen, ist nicht überraschend. Diese Patienten bedürfen einer Langzeitnachsorge und einige neue Strukturen der letzten Jahre, z. B. die Children-adolescent-young-adult(CAYA)-Einheiten an den Kliniken oder Unterstützungsangebote für junge Erwachsene der Landeskrebsgesellschaften, unterstützen diese Patientengruppe.

Da die Therapieintensität in allen Stadiengruppen auf einen ähnlichen Zielwert titriert wurde (progressionsfreies Überleben 90–95 %), erhalten die Patienten, die aufgrund der Tumorausdehnung schon bei Diagnose die stärkste Einschränkung der Lebensqualität hatten, auch die intensivste Therapie mit den höchsten weiteren Belastungen. Dass innerhalb der einzelnen Studiengenerationen der jeweils beste Studienarm nicht zu einem anderen Lebensqualitätsprofil führte als der jeweilige Vergleichsarm, ist erfreulich, denn dieser Befund bestätigt, dass ein früher Vorteil in onkologischen Outcomes nicht mit späteren Belastungen der Lebensqualität erkauft wurde.

Leider ermöglicht die aktuelle Studie keine Betrachtung der oft kontrovers diskutierten Frage, welchen Beitrag in der Behandlung des Hodgkin-Lymphoms die Chemotherapie bzw. die Strahlentherapie zu den Spätfolgen und den Veränderungen der Lebensqualität leistet, denn in keiner der inkludierten Studien wurde bezüglich des Einsatzes der Strahlentherapie randomisiert. Hier werden erst Lebensqualitätsdaten der darauf folgenden Studiengeneration interessante Einblicke liefern. Bisherige Studien zur Lebensqualität von Patienten mit Hodgkin-Lymphomen lassen jedenfalls keinen deutlichen Effekt der einzelnen Therapiemodalitäten ausmachen (Übersicht in [7]).

Wir wissen jedoch aus Langzeitdaten, dass auch die alleinige Strahlentherapie des Morbus Hodgkin (wenngleich mit höheren Dosen, größeren Zielvolumina und heute obsoleten Techniken) klinisch relevante Auswirkungen auf die Lebensqualität haben kann [8]. Inwiefern die heute im adulten oder pädiatrischen Bereich eingesetzten Strahlentherapieverfahren (z. B. 20 Gy mit Involved-node- oder Involved-site-Konzept) einen detektierbaren Einfluss auf die Lebensqualität haben, wird zukünftig von großem Interesse sein.

## Fazit

Erwachsene Patienten mit Hodgkin-Lymphom, die mit einer Kombination aus Chemotherapie und Involved-field-Strahlentherapie behandelt wurden, weisen bis zu fünf Jahre nach Therapieabschluss erhebliche Einschränkungen in den meisten Bereichen der Lebensqualität auf. Die Daten verdeutlichen den Unterstützungsbedarf dieser Gruppe von Langzeitüberlebenden. Welchen Einfluss die in den letzten zwei Jahrzehnten etablierten Modifikationen der Strahlentherapieverfahren beim Morbus Hodgkin auf Spätfolgen und Lebensqualität haben, ist aufgrund der unterschiedlichen Studiendesigns aus diesen Daten noch nicht zu entnehmen.

*Dirk Vordermark, Halle/Saale*
